# The development of child and adult care food program best-practice menu and training for Native American head start programs: The FRESH study

**DOI:** 10.1016/j.pmedr.2019.100880

**Published:** 2019-04-25

**Authors:** Susan B. Sisson, Kaysha Sleet, Rachel Rickman, Charlotte Love, Mary Williams, Valarie Blue Bird Jernigan

**Affiliations:** a Department of Nutritional Sciences, University of Oklahoma Health Sciences Center, Oklahoma City, OK, United States of America; b Center for Indigenous Health Research and Policy, Oklahoma State University, Center for Health Sciences, Tulsa, OK, United States of America; c Department of Biostatistics and Epidemiology, College of Public Health, University of Oklahoma Health Sciences Center, Tulsa, OK, United States of America

**Keywords:** Native American, Early childhood, Community-based participatory research, Nutrition, Preschool

## Abstract

New Child and Adult Care Food Program (CACFP) meal patterns and best practices were implemented nationally in 2017 to address the shift in dietary need from ensuring essential nutrient consumption to chronic disease prevention. Young American Indian (AI) children have disproportionately higher risk of chronic disease. Some AI tribes operate early care and education (ECE) programs and have the opportunity to participate in the CACFP. The purpose of this paper is to describe a CACFP best-practice menu and training developed and implemented as part of the Food Resource Equity and Sustainability for Health (FRESH) study, a community-based participatory research (CBPR) intervention implemented within ECE programs in the Osage Nation of Oklahoma. Site managers and cooks from each of the nine ECE programs attended meetings and provided investigators with feedback that shaped the best-practice menu and training. Each site participated in a three-hour training in January 2018 to discuss the best-practice menu and ways to overcome implementation barriers. Goals of the menu aimed to increase intake of fruit and vegetables and whole grains and reduce pre-fried and processed foods without increasing cook burden. Training included application activities individually and in small and large groups. Though the project is still underway, lessons learned, including the need for technical assistance, improved communication between ECE program staff and food supply vendors, and infrastructure barriers (e.g., limited space, lack of supplies) that challenge workflow, have emerged. Efforts to improve menus in rural and low-income ECE programs must consider these issues in developing feasible intervention strategies.

## Highlights

• Tribally-affiliated early care programs have unique barriers to meeting CACFP best-practice.
• Community based participatory research provides opportunity to understand barriers.
• Enhancing capacity and providing technical assistance may enhance implementation.

## 1 Introduction

Children who are overweight at the time they enter kindergarten are four times more likely to be obese at age 14, with the poorest children at greatest risk (Cunningham et al., 2014). Furthermore, children who are overweight or obese are more likely to experience significant health effects, including cardiovascular and metabolic diseases (Dietz, 1998), which can persist into adulthood (Freedman et al., 2005; Singh et al., 2008; Janssen et al., 2005). Environments where young children spend substantial time, such as early care and education (ECE) programs, are central places in which we can work to prevent obesity. By 2030, when today's preschoolers are reaching adulthood, the medical costs associated with obesity will be $48–66 billion/year (Wang et al., 2011). Primary prevention of obesity is essential to curb rising medical costs and improve quality of life (Hoelscher et al., 2013). This is particularly true in communities that have high obesity and associated health disparities, including many American Indian (AI) communities.

In Oklahoma, 36% of low-income, AI children ages 2–4 years were classified as overweight or obese in 2009 (Weedn et al., 2012). This percentage is higher than national average of 27% (Ogden et al., 2012). Young AI children have nearly double the odds of obesity (OR: 1.78; 95% CI: 1.55, 2.04) of non-Hispanic White children (Weedn et al., 2012). Studies from Oklahoma show that obesity also worsens with age; 38% (Sisson et al., 2016a) of AI children ages 3–5 years and 63% (Dennison et al., 2015) of AI children ages 7–13 years are overweight or obese. As the majority of U.S. children up to 6 years old regularly spend time in ECE programs (Redford et al., 2017) and ECE providers are required to feed children nearly two-thirds of their daily nutrient needs (Oklahoma Department of Human Services, 2016), the importance of ECE programs cannot be underestimated. Studies have shown that ECE program policies and their social and physical environments influence child physical activity (Trost et al., 2010; Tonge et al., 2016) and dietary intake (Erinosho et al., 2011; Kharofa et al., 2015; Gubbels et al., 2010; Hughes et al., 2007; Anundson et al., 2018; Lessard and Breck, 2015; Sisson et al., 2016b). However, there is great variation in quality of health-promoting practices and policies across ECE programs (Swyden et al., 2017; Schwartz et al., 2015; Benjamin Neelon et al., 2012).

Research within ECE programs in AI communities is scarce. We conducted ECE program observation within 11 tribally-affiliated ECE programs in Oklahoma. As part of this process, we assessed ECE programs and policies that promote active play and healthy food options, including fruits and vegetables on the menu. We also measured dietary intake and physical activity in AI children attending the tribally-affiliated ECE programs. The AI children that attended a program with opportunities for active play and healthier meal options had 9% lower odds of being overweight or obese compared with AI children who attended a program without such policies in place (Sisson et al., 2016a). To our knowledge, no other studies have examined ECE environments in AI communities.

The National Academies of Medicine (Institute of Medicine, 2011) and the American Academy of Pediatrics (*Am. Acad. Pediatr.*, 2005) have described ECE programs as opportune environments in which to establish healthy behaviors to prevent obesity (Story et al., 2006), and outlined strategies that ECE programs can take to create more healthful and less obesogenic environments. These strategies include the provision of high-quality, nutritionally dense meals comprised of vegetables and fruits. Additionally, ECE providers serving low-income children can participate in the Child and Adults Care Food Program (CACFP), which reimburses qualifying food costs (United States Department of Agriculture, 2018) and is associated with increased access to nutritious foods (Ritchie et al., 2012; Korenman et al., 2013). However, there are variations in the fidelity with which the CACFP is implemented, which may compromise overall nutritional quality (Schwartz et al., 2015; Monsivais et al., 2011), and leaves room for improvement.

In fall 2017, new CACFP meal patterns and best-practices were implemented nationally. The purpose of the updated CACFP meal patterns was to better align them with the Dietary Guidelines for Americans and respond to dietary need shift from ensuring consumption of essential nutrients to prevention of chronic diseases, including obesity (United States Department of Agriculture, 2018; United States Department of Agriculture, 2017). The new meal pattern emphasized nutrient density and reduced added sugars, saturated fat, and sodium (United States Department of Agriculture, 2018). A Health Impact Assessment of the new meal pattern suggested it should improve the nutritional quality of meals in those programs participating in the CACFP (Lindberg and Majumdar Narayan, 2017). It also emphasized the need for educational materials and resources, such as training for diverse populations, to achieve maximum implementation and benefit of the new meal patterns and best practices (Lindberg and Majumdar Narayan, 2017).

The Food Resource Equity and Sustainability for Health (FRESH) study aims to increase vegetable and fruit consumption and reduce body mass index and hypertension among AI families. Included as part of the study was a CACFP best-practice menu and teacher training to increase vegetable and fruit servings and reduce added sugars, saturated fat, fried and pre-fried foods, and sodium within the Osage Nation ECE program menu. The purpose of this paper is to describe the community-based participatory research orientation used to develop and implement this CACFP best-practice menu and the training of service providers and teachers to implement this menu within ECE programs in the Osage Nation of Oklahoma. We present lessons learned for future interventions to improve the health of AI children within ECE programs.

## 2 Methods

### 2.1 Community context and partnership

The Osage Nation, located in Northeastern Oklahoma, occupies the state's only federally recognized reservation, which is coterminous with Osage County. The total tribal membership is 11,394, of whom 5682 reside on the reservation (United States Census Bureau, 2011). The poverty rate in Osage County is 23% (United States Census Bureau, 2011). The most recently published Behavioral Risk Factor Surveillance Study data showed that 77% of Osage tribal members were overweight and, of those, 36% were obese (Bursac and Campbell, 2004). The Osage Nation operates a number of programs and services for AI families, including a Community Health Representative Program, a Community Health Department that offers free health education and fitness classes, and the Food Distribution Program on Indian Reservations (FDIPR), a United States Department of Agriculture program that provides canned and packaged foods to ~85,000 tribal participants per month with limited access to the Supplemental Nutrition Assistance Program (SNAP) (Pindus et al., 2016).

In addition to these public health and social service programs, the Osage Nation operates nine ECE programs: four Head Start programs; four Wah-Zha-Zhi Early Learning Academies (WELAs); and one Osage Language Immersion school. These programs are located in the four Osage communities of Skiatook, Fairfax, Hominy, and Pawhuska. The oldest of the programs, the Osage Nation Head Start programs, began operations in 1979 and continued to add locations until 1985, with enrollment ranging from 19 to 95 children across sites. The WELA facilities opened approximately five years ago and serve between 12 and 26 children across sites. Finally, the Osage Nation Language Immersion School, opened in 2015. There are 34 children enrolled in this program. While all but one of these ECE programs officially participates in the CACFP, they all share one central menu planning team. A registered dietitian oversees the menus. Although the programs are distinct in terms of their educational approaches (i.e., about half of the programs include an additional emphasis on Osage language and culture), they are all located within the Osage Nation and are also similar in demographic makeup.

The FRESH study partnership began in 2013 and comprises a multisector group of representatives from various tribal sectors, in addition to university partners. The tribal-university partnership surveyed diverse stakeholders across Osage Nation to assess the feasibility of and community readiness to implement evidence-based strategies to improve the Osage Nation food environment. Participants identified “increasing the availability of healthy foods through tribally owned and operated venues” as the best strategy for implementation, and their scores indicated the “preplanning” stage of readiness (Jernigan et al., 2016). In response, the tribal-university partnership developed the FRESH study.

### 2.2 FRESH study design

The FRESH study was funded in 2016 and is led by the multisector tribal-university study executive committee. The study is a randomized, wait-list controlled trial to evaluate the impact of a gardening and education intervention on vegetable and fruit intake and food insecurity, BMI, and blood pressure among a cohort of AI families (*n* = 250 families) over a 6-month period. The executive committee developed the following key components of the FRESH study intervention: 1) a 15-week culturally relevant gardening and healthy eating curriculum implemented in the nine Osage Nation ECE programs; 2) web-based weekly workshops for parents to support healthful parenting, sleep, nutrition, and physical activity routines for children; 3) monthly in-person meetings to build local community capacity to create healthier community food environments; and 4) the development and implementation of a comprehensive CACFP best-practice menu and training for the Osage ECE programs. Here we describe the CACFP best-practice menu and training component.

### 2.3 Development of CACFP menu and training for the Osage ECE programs

During the FRESH study planning process, members of the executive committee as well as an extended team of ECE program teachers and site managers identified the need for a best-practice menu and training for the Osage ECE staff as an additional component to be added to the FRESH study. To address this request, university partners consulted a nutritional researcher (first author of this manuscript) to work with the study executive committee. The first step in this process was to assess the ECE program goals for their menus and training needs. This assessment involved several structured meetings beginning August 2017 through January 2018, when the final menu and training were launched. The meetings included ECE program teachers, site managers, and food preparation staff, and focused on the new CACFP meal pattern changes, community partner food preparation process, vendors, storage, and educational, staff and infrastructure needs.

These meetings identified several key issues. These concerns were the limited availability and quality of local grocers across communities and limited availability of vendors, which in turn restricted food access. The group also identified internal factors that focused primarily on local infrastructure needs, such as budget stability, site storage concerns, staff and time availability, and variability in the size of the ECE programs within the communities. For example, although the ECE programs used the same vendor, the vendor delivered twice per week to larger ECE program sites and once per week to smaller ECE program sites, depending on location. Occasionally produce arrived spoiled, and because not all the communities had access to grocery stores with affordable, quality produce in the amount needed, substitution of spoilt produce was not always possible. These factors contributed to the menu variation across ECE program sites.

In addition, staff reported that some of the sites had extremely small food preparation and storage areas that precluded larger delivery of non-shelf stable foods (e.g., fresh produce). One site reported not having any food preparation or storage space, and all food was prepared at another site and delivered, which placed double the cooking burden on the food preparation staff at the larger site. Some sites had limited food preparation staff and had staff working in the kitchen as well as floating between classrooms or operating as the site director. The concern raised was a lack of time available for any scratch or partial scratch food preparation. A reliance on processed foods that were single-serve or that could be quickly heated and served was considered a necessity.

These concerns highlighted the need for accompanying changes to the local reservation food environments and ECE program infrastructures. While the infrastructure needs have been reported by diverse programs elsewhere (Robert Wood Johnson Foundation, 2013), the use and reliance on the FDPIR commodity food program provided by the U.S. government is unique to AI communities (Pindus et al., 2016). Approximately half of the ECE programs were required to incorporate FDPIR commodity foods in their menus to augment existing foods. The provision of these foods and exactly what is available and provided to these ECE programs can vary greatly from month-to-month, and can be unpredictable. This factor was also an important source of variability for the programs.

The university partners incorporated all of these concerns into a draft best-practice menu and training protocol. The draft menu and training protocol were provided to each of the nine program directors and food preparers. A conference call was held in November 2017 to review the documents with community partners and incorporate additional feedback. University partners also inquired about aspects of the menu that were considered favorable or raised concerns with implementation. These concerns were addressed in a final best-practice menu and training protocol that was delivered and presented at the 3-hour training held in December 2017 in Hominy, Oklahoma. The ECE directors, teachers, and food preparation staff for each program were invited to attend this training. There was representation from all sites at the training.

## 3 Results

### 3.1 Final best-practice menu

**Fig. 1:**
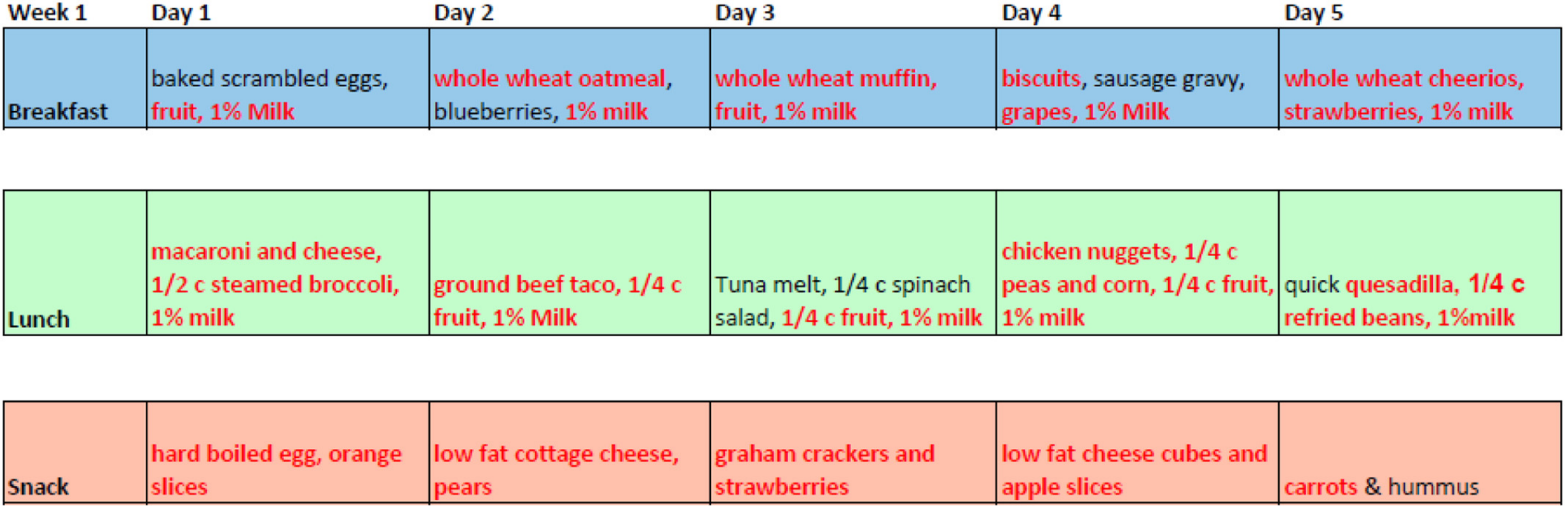
Example week of best-practice menu prepared for rural tribal ECE. *Red text indicates a food retained from the original 6-week cycle menus provided by the community. (For interpretation of the references to color in this figure legend, the reader is referred to the web version of this article.)

The six-week cycle best practice menu developed for the Osage Nation ECE programs is shown in Table 1. The best-practice menu goals aimed to increase intake of fruit, vegetables, and whole grains, and reduce pre-fried and processed foods. Several steps were taken to ensure that the best-practice menu addressed infrastructure concerns raised during the assessment process. First, research personnel contacted the food vendor for the Osage Nation ECE programs, accessed their online catalog, and verified that all food and ingredients for the new menu would be available. Second, to maintain quick and easy food processing, most of the existing recipes were retained and modified to enhance nutritional quality. For example, a familiar recipe of quesadillas was reviewed and revised to include more vegetables and whole grain tortillas. Pre-fried and breaded frozen chicken was replaced with pre-grilled frozen chicken. Similarly, pre-fried meats and vegetables were replaced with fresh or frozen varieties that could be steamed or baked. The retained recipes were highlighted in red. Recipes for all new foods were included. Any new recipes were examined for complexity and number of ingredients. A one-week example of the best-practice menu is shown in Fig. 1. A summary of the differences between the original and best-practice menu is shown in Table 2.

**Table 1:** Best-practice menu goals.

1. Include fruits and vegetables as snacks
2. Eliminate all juice
3. Include vegetable subgroups throughout the week
4. Serve only lean meats, nuts, and legumes
5. Serve meals family-style
6. Serve 2 servings of whole grain food per day
7. Eliminate all sugary beverages, including juice, soda, lemonade, and juice drinks
8. Limit pre-fried foods to no more than 1 time per week

**Table 2:** Differences from original to best-practice menu for example week.

1. Adding eggs in 1 breakfast
2. Adding fresh or frozen blueberries in 1 breakfast
3. Adding tuna melt sandwich at 1 lunch
4. Adding spinach salad at 1 lunch
5. Adding bell peppers at 1 lunch
6. Replacing quesadilla recipe
7. Adding hummus at 1 snack
8. Removing milk component at snack in lieu of fruit or vegetable (can still be served without reimbursement)

Several infrastructure concerns could not be addressed. These concerns were the limited access to grocery stores, vendor delivery frequency, and limited space for food preparation and storage. These concerns were raised with parent groups as part of a separate component of the study. In addition, meal planning strategies to reduce storage space and waste (i.e., repetition of ingredients during the course of the week) were built into the best-practice menu in efforts to minimize staff burden.

### 3.2 Training

A tailored training on the implementation of the best-practice menu and how to implement it amidst the challenges and barriers identified by the community took place in December 2017 at a tribal education facility. The training program itself was based on adult learner needs (Norris, 2003) and previously identified needs in knowledge (Sisson et al., 2018; Sisson et al., 2017; Kracht et al., 2019). The program was broken into smaller sections (i.e., six modules) with heavy emphasis on application and integration activities. Activities were conducted individually, in small groups, and in large groups to offer variety and team building. We integrated food preparation staff personal experience, knowledge needs and desires, and the CACFP best practices into this interactive 3-hour training. Throughout the training, several opportunities were provided to develop a specific goal and action plan to make changes at their site. Goals were organized in a SMART (specific, measurable, attainable, realistic, time-sensitive) format to encourage participants to think through the goal and provide a time frame and action steps to get started. Topics of training included: 1) why meet best practices; 2) best practices in food preparation; 3) strategies for easier menu planning; 4) understanding food labels; 5) easy recipe modifications; and 6) explanation and discussion of the best-practice menu. Objectives of each module are included in Table 3. Each participant received a binder with a handout for each module with key information highlighted, an activity sheet, space for notes, and additional resources. Additional handouts, including those about vegetable subgroups, setting SMART goals, graphics on how and where to store different types of foods to reduce food waste, and nutrition labels, were also included. A color copy of all slides was included in the participant binder.

**Table 3:** Best-practice menu training curriculum content and activities.

Module	Module content description	Activities
Why meet best practices (30 min)	• Identify CACFP best practices and their importance for nutrient intake • Identify barriers to best practices and describe solutions	• Large Group Activity – Identify which frozen foods from pictures are fried, baked, or grilled • Individual Activity – Identify barriers and solutions to following best practices • Individual Activity – Set a SMART goal to start a best practice
Food preparation best practices (30 min)	• Understand the best practices for handling food • Understand why best practices are important • Identify strategies to implement best practices with limited resources	• Small Group Activity – Identify foods from a 1-week menu that can be prepped in bulk ahead of time • Small Group Activity – Identify items from a menu that could be made from scratch • Individual Activity – Set a SMART goal to incorporate scratch preparation
Menu planning (20 min)	• Learn steps for effective menu planning • Understand how to make a menu that is appealing to children	• Large Group Activity – Identify qualities from pictures that make plates look appealing and how to improve the visual appeal • Small Group Activity – Choose two meals from current menu and identify appealing qualities and substitutions to increase appeal • Individual Activity – Set a SMART goal to improve current menu-writing process
Food labels (20 min)	• Learn components of a nutrition label • Recognize how to interpret food labels to compare products • Understand health claims of food packages	• Individual Activity – Identify certain nutrients listed on a nutrition facts label based on different serving sizes • Individual Activity – Match package health claims with required criteria
Recipe modifications (30 min)	• Recognize why recipes would be modified • Identify healthy ingredient and preparation substitutions • Learn how to modify recipes	• Individual Activity – Complete a healthy cooking quiz, reviewed at the end of the module • Small Group Activity – Identify healthy modifications that could be made to an predetermined recipe • Small Group Activity – Choose 1–2 recipes from current menu and identify healthy modifications that could be made • Individual Activity – Set a SMART goal for a healthy modification within the current menu

### 3.3 Best-practice menu implementation

The best-practice menu was developed for implementation beginning January 2018. Process evaluation data assessing the implementation of the menus, such as observation of weekly menu, at each of the nine sites was collected by university staff. University staff collected information from the cooks at each site at five different time points from fall 2017 to fall 2018. Staff made copies of the menus that the cooks were using and recorded what was actually served, ingredients, quantity, and preparation methods for a one-week time period (always week 5 of the cycle). The impact of the best-practice menu and training, along with complete FRESH outcomes, will be reported in future manuscripts. Of the nine ECE Programs, four implemented the best-practice menu in spring 2018, as determined by examining the working menus at each program. All wait-list programs and one intervention Head Start program did not adopt the best-practice menu.

## 4 Discussion

This paper describes the development of a CACFP best-practice menu and additional training for food preparation staff in ECE programs within the Osage Nation. The study process revealed challenges to meeting CACFP best practices in rural, tribal ECE environments. These challenges included limited access to healthy foods, staffing shortages, and limited food preparation space. While these challenges have been reported in other ECE program settings, both rural and urban ECE (Robert Wood Johnson Foundation, 2013), reliance on the FDPIR to augment ECE program foods is a unique aspect of tribally-affiliated ECE programs that may contribute to variations in food quality and availability in tribal ECE programs. Additionally, our own research has shown that foods sold in stores on or near reservations cost more than do the same foods sold in neighboring non-reservation stores (Jernigan et al., 2012; McLaury et al., 2016). These barriers may also hinder the availability of healthy foods in tribal ECE program menus, and warrant further investigation.

This study is, to our knowledge, the first to utilize a CPBR approach to develop and implement a CACFP best-practice menu and training for ECE program staff. Previous studies to prevent obesity within ECE programs have focused primarily on individual-level health behavior strategies with children, and less commonly included staff training and technical assistance for menu changes (Sisson et al., 2016b; Copeland et al., 2012; Lindsay et al., 2015; Rosenthal et al., 2013). Three studies conducted in Australia (Finch et al., 2019; Yoong et al., 2016; Bell et al., 2015), one in New York (Williams et al., 2002), and one in California (Alkon et al., 2014) included training for cooks that went beyond menu modifications. Three studies included a five-to-eight-hour training with cooks, which resulted in enhanced menu nutritional quality (Bell et al., 2015), reduced children's fat intake (Williams et al., 2002), and enhanced nutrition practices and policies (Alkon et al., 2014). However, another study that included a full-day training for cooks did not result in menu changes (Finch et al., 2019). One study included educational materials tailored for ECE cooks with improved intent to use nutritional guidelines, but was insufficient to enhance menu quality (Yoong et al., 2016). Qualitative work conducted by our own team indicates that teachers and providers desire to know more about child nutrition and health behaviors than the basic guidelines for menu development provided to them during routine trainings (Sisson et al., 2018; Sisson et al., 2017; Kracht et al., 2019).

Though the FRESH study is still underway, lessons learned have already emerged. During the menu and training implementation, we identified the need to include on-site technical assistance at ECE programs to work on goals for efficiency in kitchen workflow and food preparation. Tribally-affiliated ECE, especially in rural areas, have food access and procurement barriers. Understanding of and potential partnership with supply chain, vendors, local grocers, and possibly farmers are important for long-term success in providing healthier nutrition in these programs. Collaboration with the site manager and cook is essential to fully understand perceived and actual barriers to provide training that can provide necessary background rationale for nutrition recommendations, as well as beneficial and productive activities and hands-on training to effect change. The various levels of capacity and infrastructure within an ECE program dictates, to some degree, their ability and desire to strive towards best practices. Even though all nine ECE programs are operated through Osage Nation, Head Start programs have federally mandated performance standards regarding nutrition and physical activity with which they must comply (United States Department of Health and Human Services, 2011). Enrollment and care capacity may also be an important consideration. In the smaller programs, fewer staff are employed. This may result in last-minute meal preparation by staff whose primary role is not food preparation, or the necessity to prepare food at one site and transport the food to another site.

The challenges identified in this CBPR study can be used by other community-university partnerships to develop similar programs with rural ECE programs, including tribally-affiliated ECE programs, indicating external validity of this process. University partners worked closely with the tribal and ECE program representatives to develop a menu and training that advanced health goals while addressing community-identified barriers with high rigor to the CBPR process enhancing internal validity. Limitations include minimal data on the effect of the menu and training. However, given that the purpose of this paper is to share the process of developing a menu and training, using CBPR orientation, with a tribal community, discussion of the intervention impact is not needed. Future research currently underway will explore facilitators and barriers to improving ECE program menus based on program characteristics and tailoring menu change strategies for these unique environments. We hypothesize that barriers would include vendor delivery schedule, kitchen size and food preparation space, and staffing demands. The present study will yield important findings in the efforts to improve tribal food environments and inform policy makers about the efficacy of menu change interventions in improving child dietary intake and the feasibility of implementing such efforts in diverse ECE program contexts.

## Contributor statement

V. Blue Bird Jernigan conceptualized and supervised the study and assisted with writing. S. Sisson supervised and guided development of the best-practice menu and training, and led the manuscript writing. K. Sleet and R. Rickman developed the best-practice menu and training program, and assisted with writing. C. Love assisted with data collection, data analysis, and writing. M. Williams assisted with conceptualization, data presentation, writing, and interpretation.

## Acknowledgments/COI

This study was funded by the National Institute on Minority Health and Health Disparities (R01MD011266). The funding agency did not participate in the study design, data collection, analysis, decision to publish, or preparation of the manuscript. All authors approve of the final version. This work has not been published or presented elsewhere. No authors have a conflict of interest. We thank the members of the Osage Nation.

## Human participant protection

The study was reviewed and approved by the Institutional Review Board of the University of Oklahoma Health Sciences Center.
